# Angiotensin-Receptor-Associated Protein Modulates Ca^2+^ Signals in Photoreceptor and Mossy Fiber cells

**DOI:** 10.1038/s41598-019-55380-8

**Published:** 2019-12-23

**Authors:** Rene Barro-Soria, Alejandro Caicedo, Herbert Jägle, Laura Merkel, Na Zhao, Gabriel Knop, Kaspar Gierke, Andrea Dannullis, Hayo Castrop, Johann Helmut Brandstätter, Frank Kirchhoff, Andreas Feigenspan, Olaf Strauß

**Affiliations:** 1 Experimental Ophthalmology, Eye Hospital, University Medical Center Regensburg, 93053 Regensburg, Germany; 2 Department of Medicine, University of Miami Miller School of Medicine, Miami, FL. USA; 3 Division of Endocrinology, Diabetes and Metabolism, Department of Medicine, University of Miami Miller School of Medicine, Miami, FL. USA; 4 Department of Biology, Division of Animal Physiology, University of Erlangen-Nürnberg, 91058 Erlangen, Germany; 5 Department of Molecular Physiology, University of Saarland, 66421 Homburg, Germany; 6 Institute of Physiology, University of Regensburg, 93053 Regensburg, Germany; 7 Experimental Ophthalmology, Department of Ophthalmology, Charité – Universitätsmedizin Berlin, a corporate member of Freie Universität, Humboldt-University, the Berlin Institute of Health, 13353 Berlin, Germany

**Keywords:** Neuroscience, Physiology

## Abstract

Fast, precise and sustained neurotransmission requires graded Ca^2+^ signals at the presynaptic terminal. Neurotransmitter release depends on a complex interplay of Ca^2+^ fluxes and Ca^2+^ buffering in the presynaptic terminal that is not fully understood. Here, we show that the angiotensin-receptor-associated protein (ATRAP) localizes to synaptic terminals throughout the central nervous system. In the retinal photoreceptor synapse and the cerebellar mossy fiber-granule cell synapse, we find that ATRAP is involved in the generation of depolarization-evoked synaptic Ca^2+^ transients. Compared to wild type, Ca^2+^ imaging in acutely isolated preparations of the retina and the cerebellum from ATRAP knockout mice reveals a significant reduction of the sarcoendoplasmic reticulum (SR) Ca^2+^-ATPase (SERCA) activity. Thus, in addition to its conventional role in angiotensin signaling, ATRAP also modulates presynaptic Ca^2+^ signaling within the central nervous system.

## Introduction

The retina expresses its own intraretinal renin-angiotensin-system (RAS) that includes all metabolites and proteases such as renin or angiotensin-converting enzyme^1^. One of the main effectors of RAS is angiotensin-2 (AngII or Ang1–8). Perturbations in the retinal RAS change the waveforms of the electroretinogram, which suggests a role of RAS in the signal processing of the retina^2^. Thus, cells in the retina that express AngII receptors form the signal processing network that is regulated by RAS. AngII-sensitive cells might include a specialized subset of bipolar cells and, better investigated, a subset of ganglion cells^1^. Binding of AngII to its receptor angiotensin-2-receptor type-1 (AT1R) increases intracellular free Ca^2+^, thereby suggesting that AngII might affect the electrical activity of retinal neurons^1^. The angiotensin-receptor-associated protein (ATRAP) is known to interact with and modulate the cytosolic side of AT1R in the systemic RAS network^3,4^. Not surprisingly, the vast majority of studies about ATRAP’s functional role are those exclusively related to the modulatory effect of ATRAP on the angiotensin-receptor signaling pathway^5^. However, in the retinal pigment epithelium (RPE) ATRAP has also been shown to play a role in the Ca^2+^ store-dependent Ca^2+^ signaling, as well as in modulating AT1R-evoked Ca^2+^ through activation of TRPV2 channels^6^. More recently, Mederle *et al*.^7^ showed that ATRAP physically binds to and stimulates the cardiac sarcoendoplasmic reticulum Ca^2+^-ATPase 2a (SERCA2a). In addition, ATRAP interacts with other molecules such as calcium-modulating cyclophilin ligand in the endoplasmic reticulum to regulate intracellular Ca^2+^ fluxes^8^. While investigating the role of ATRAP in the retina, we found that ATRAP was abundantly present in the RPE and in the outer plexiform layer (OPL) of the retina^6^, a region that contains photoreceptor terminals and postsynaptic horizontal and bipolar cell dendrites. RPE cells from ATRAP knockout mice (*Atrap*^-/-^) show decreased amplitudes of AngII evoked Ca^2+^ signals^6^. Surprisingly, the role of ATRAP in the photoreceptor synapse has not been investigated yet. Since ATRAP is a protein that modulates intracellular Ca^2+^ signaling and because of its localization in the OPL, we here tested the hypothesis that ATRAP is involved in Ca^2+^ signaling at the photoreceptor synapse and potentially in other ATRAP-expressing neurons in the central nervous system.

Ca^2+^ signaling in synapses contributes to triggering depolarization-dependent neurotransmitter release^9,10^. Depolarization results by action potentials that arrive at the synaptic terminals or in the photoreceptor by shut-down of the light transduction cascade in the outer segment during transition from light to darkness. The depolarization activates voltage-dependent Ca^2+^ channels that generate an influx of extracellular Ca^2+^ into the synapse, which represents the trigger for neurotransmitter release. Store-operated Ca^2+^ entry represents an important regulation of intracellular free Ca^2+^ at synaptic terminals. Store-operated Ca^2+^ entry results from Ca^2+^-dependent depletion of cytosolic Ca^2+^ stores which in turn activates an additional type of Ca^2+^ channel in the plasma membrane as a mechanism to enhance the Ca^2+^ signal^11–19^. At the presynapse of cerebellar interneuron-Purkinje cell junctions, ryanodine-dependent release of Ca^2+^ from cytosolic stores leads to large miniature inhibitory postsynaptic currents in Purkinje cells^17–19^. In the rod synapse, Ca^2+^ enables recruitment of non-ribbon glutamate release during sustained depolarization^16,20–24^. The increase in presynaptic Ca^2+^ concentration is terminated by the activity of the plasma membrane Ca^2+^-ATPase and the uptake of Ca^2+^ into cytosolic Ca^2+^ stores by the activity of sarcoplasmic Ca^2+^-ATPase (SERCA2)^15,23,25–29^.

Given the expression of ATRAP and SERCA2 in the photoreceptor cells, studying the role of ATRAP in the retina provides insights into RAS-mediated retinal function, particularly in the photoreceptor synapse. Thus, we aimed to understand the function of ATRAP and its possible interaction with SERCA in the photoreceptor synapse. To examine the role of ATRAP, we performed molecular, anatomical, and *in vitro* physiological studies. We found that ATRAP localizes to the synapses of both rod and cone photoreceptors in the retina and also showed abundant expression in other regions of the central nervous system such as the cerebellum. Surprisingly, we found ATRAP expression in the OPL, a region that does not express AT1R, therefore indicating a potentially complete new role for ATRAP, in synapses. Ca^2+^ imaging experiments demonstrated that in the retina and the cerebellum ATRAP functions as a synaptic protein that affects the magnitude and time course of the presynaptic Ca^2+^ signal. Our data demonstrate a novel physiological function for ATRAP in the presynaptic terminals of photoreceptors and mossy fibers of the cerebellum, where it modulates depolarization-evoked Ca^2+^ signals.

## Results

### Expression of ATRAP in the mouse retina

**Figure 1:**
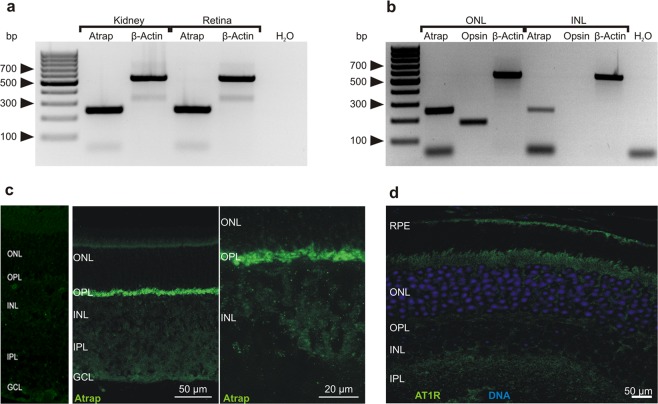
ATRAP is expressed in the outer plexiform layer of the mouse retina. (**a**) RT-PCR performed with retinal tissue shows robust expression of ATRAP mRNA. mRNA from kidney tissue served as control. (n = 3 independent tissue samples for kidney and retina). (**b**) RT-PCR performed on laser captured ONL and INL shows expression of ATRAP mRNA. Opsin specifically expressed in the ONL but not in the INL served as control. (n = 3 for ONL and INL). (**c**) Confocal image of a vertical section of a mouse retina shows ATRAP immunostaining (green) mainly in the OPL. Left panel shows ATRAP immunostaining in *Atrap*^*−/−*^ mouse retina and the middle/right panel shows ATRAP immunostaining in *Atrap*^*+/+*^ mouse retina. (**d**) Angiotensin II-receptor type-1 (AT1R) immunostaining shows localization in the retinal pigment epithelium and inner segments of the photoreceptors but not in the outer plexiform layer. Outer nuclear layer (ONL), outer plexiform layer (OPL), inner nuclear layer (INL), inner plexiform layer (IPL) and ganglion cell layer (GCL), retinal pigment epithelium (RPE). Immunohistochemistry was carried out with two slices from two different animals.

The first sets of experiments served to verify the expression of ATRAP in the retina and to establish its localization in specific cell types. We detect mRNA expression of ATRAP by RT-PCR in whole mouse retina (Fig. 1a). Using laser capture microdissection of the mouse retina, we find that ATRAP mRNA is present in both the outer and inner nuclear layer (Fig. 1b). ATRAP immunoreactivity is strong in the outer plexiform layer of the wild type (*Atrap*^*+/+*^) mouse retina (Fig. 1c, middle and right panel) but not in the retina of mice lacking ATRAP (Fig. 1c left panel; *Atrap*^*−/−*^)^6^. Because ATRAP has been reported to modulate angiotensin receptor signaling, we examined the expression of angiotensin receptor (AT1R) in the retina. As previously reported^30^, weak AT1R immunostaining by an antibody that gives no signal in AT1R knockout mouse kidney is seen in the retinal pigment epithelium but is absent from the outer plexiform layer (OPL) (Fig. 1d), suggesting that in the OPL ATRAP is not involved in conventional angiotensin receptor signaling. Therefore, we use the retina to assess the function of ATRAP without the angiotensin-signaling mediator present.

### ATRAP is not expressed in horizontal cells, rod and ON cone bipolar cells

**Figure 2:**
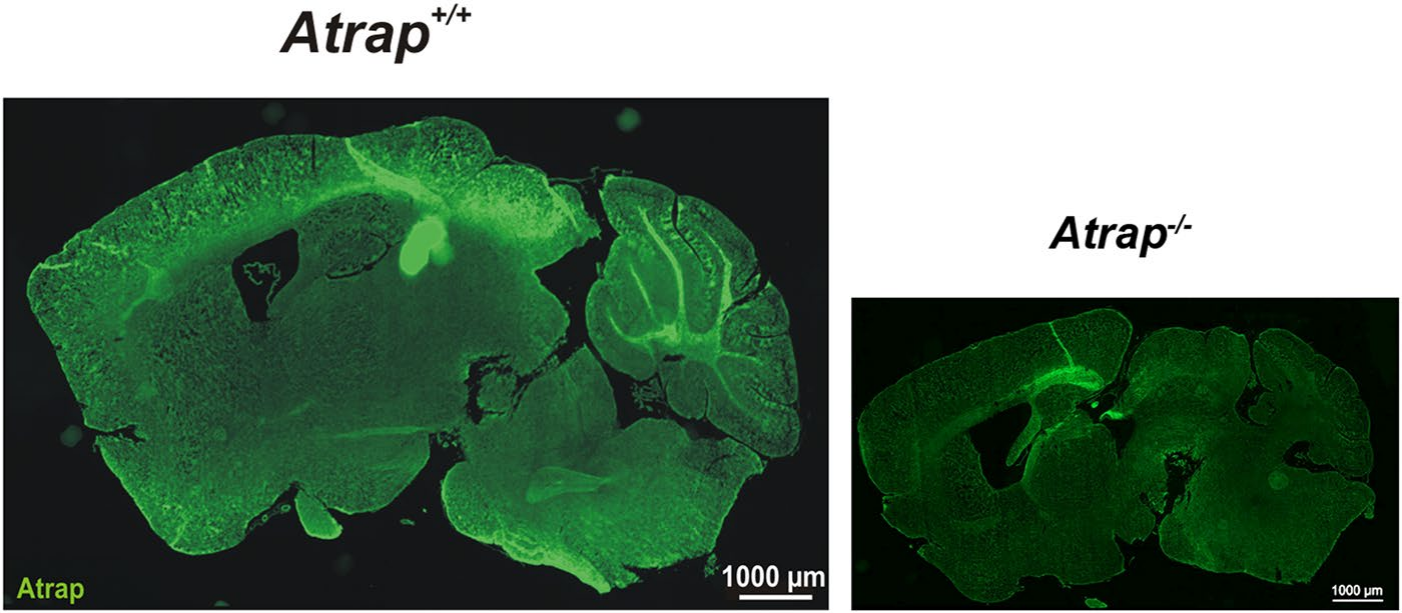
ATRAP expression in the brain. Confocal image showing ATRAP staining in the cerebral cortex and the cerebellum of *Atrap*^*+/+*^ mouse brain (left panel; right panel *Atrap*^*−/−*^ mouse control). Note that ATRAP is readily expressed in the cerebellum, neocortex and, weaker, in hypothalamic areas.

**Figure 3:**
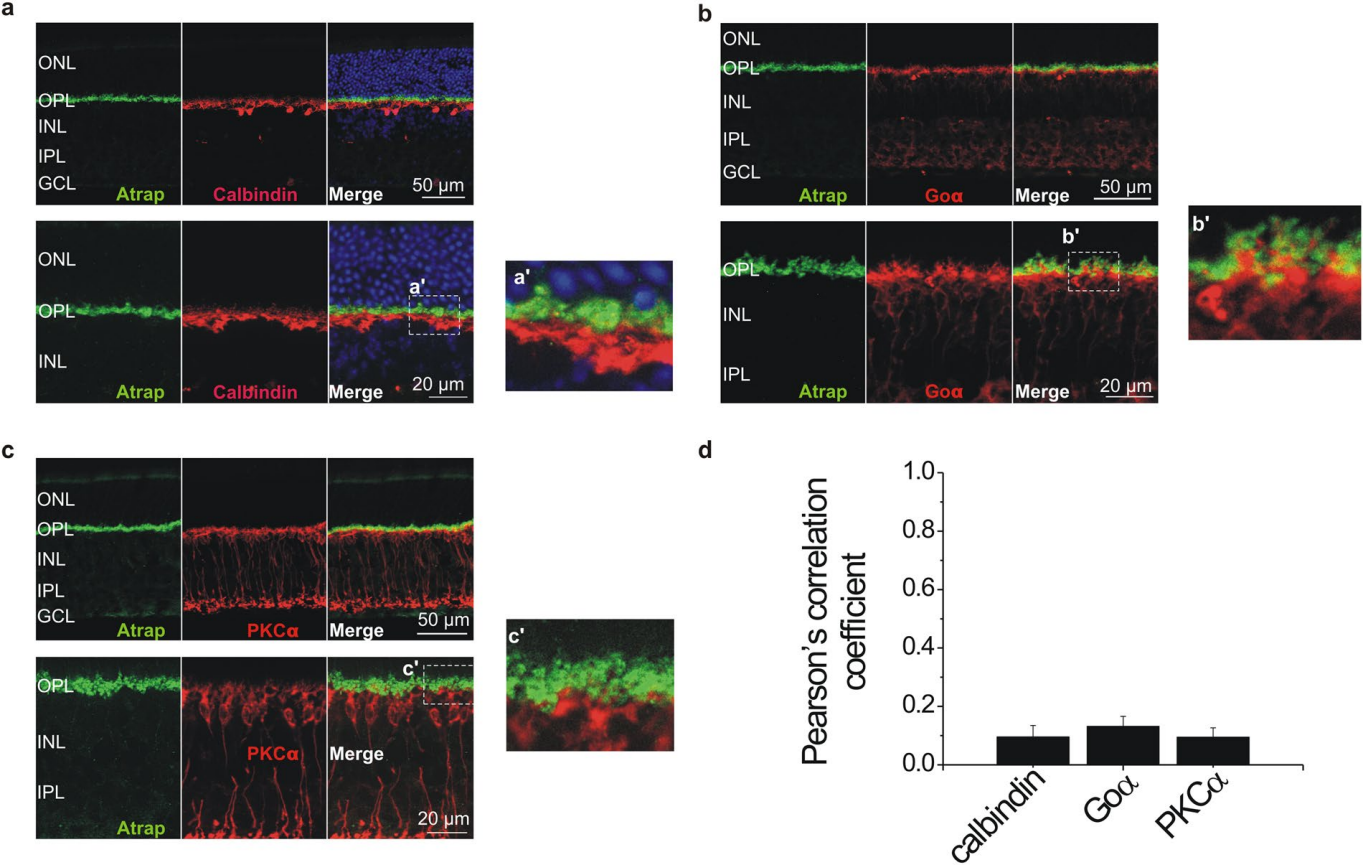
ATRAP is not expressed in horizontal cells and ON bipolar cells. Confocal images of vertical sections of mouse retinae (upper panels) and OPL (lower panels). ATRAP immunostaining (green) did not overlap with calbindin (red in **a**), Goα (red in **b**) or PKCα (red in **c**) in the OPL, indicating that ATRAP is not expressed in horizontal cells and in ON cone and rod bipolar cells. Images on the right (**a’–c’**) are higher magnifications of retinal regions delimited by the boxes shown in (**a**–**c**). Outer nuclear layer (ONL), outer plexiform layer (OPL), inner nuclear layer (INL), inner plexiform layer (IPL), ganglion cell layer (GCL). (**d**) Bar graph showing the thresholded Pearson’s correlation coefficient values for colocalization of ATRAP immunostaining with calbindin, Goα and PKCα. Large values indicate stronger colocalization, whereas values close to 0 imply lack of colocalization. Data are mean ± SEM, n = 4 in each group.

Our results show that ATRAP is robustly expressed in the OPL, which is composed of a complex network of photoreceptor terminals and horizontal and bipolar cell dendrites. We therefore performed cell type-specific immunohistochemistry to localize ATRAP within the OPL. Using the retina of the *Atrap*^*−/−*^ mice we verified that the antibody used to detect ATRAP does not produce false positive signals (Fig. 1c, left panel; Fig. 2 right panel). We find that ATRAP was almost not detectable in horizontal cells, which are identified by calbindin immunostaining^31^ (Fig. 3a,d). ATRAP is also barely detectable in rod bipolar cells identified by Goα immunostaining^32^ and PKCα immunostaining^33^ and in ON cone bipolar cells, identified by Goα immunostaining^32^ (Fig. 3b–d).

### ATRAP localizes in ribbon synapses of photoreceptor cells

**Figure 4:**
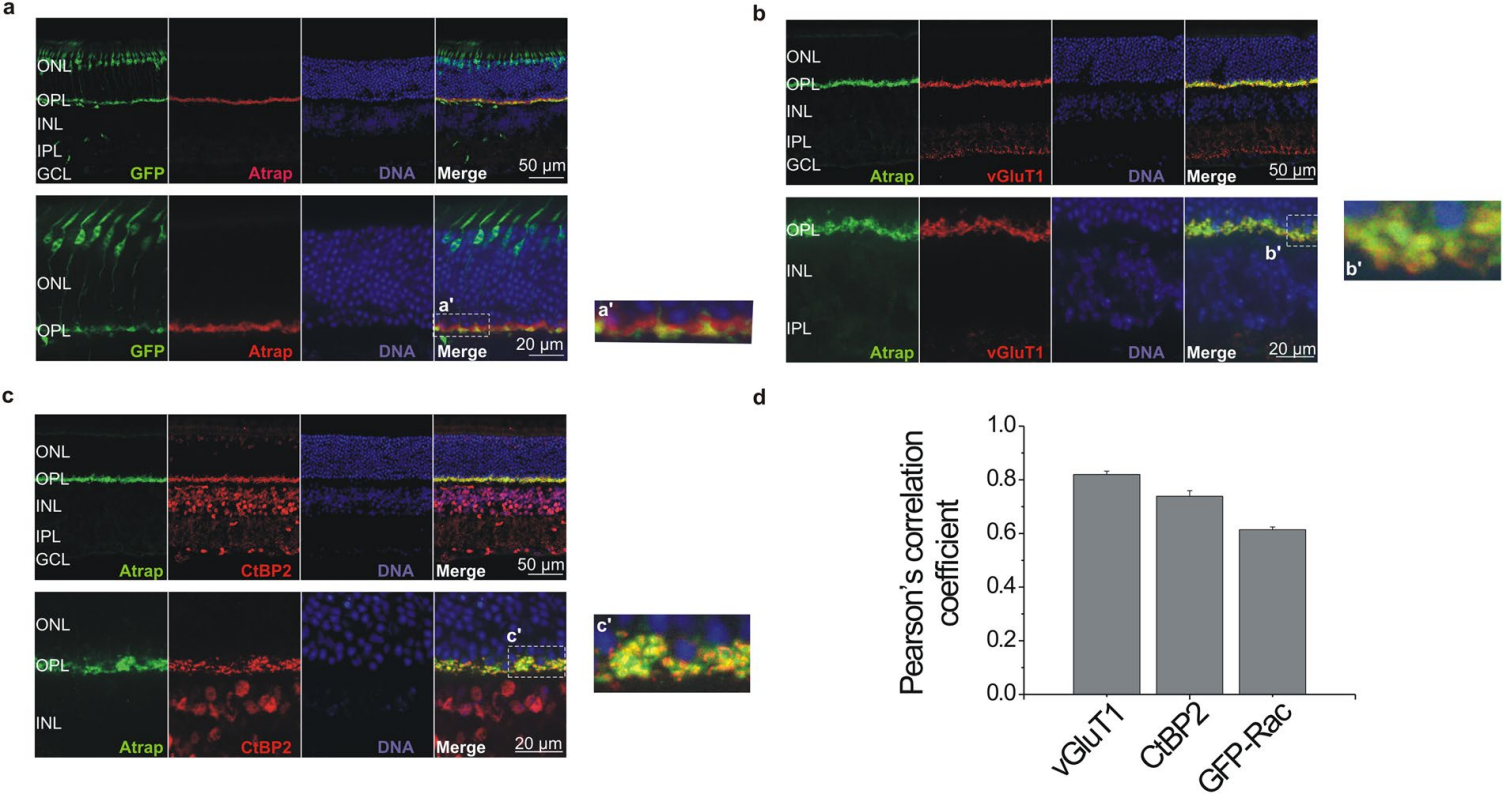
ATRAP is localized in the ribbon synapses of rod and cone photoreceptors. Confocal images of vertical sections of mouse retina (upper panels) and OPL (lower panels). (**a)** Retinae from transgenic mouse line (Rac3-eGFP) in which cone photoreceptors express EGFP (green) show robust colocalization of ATRAP (red). (**b**) ATRAP (green) and vesicular glutamate transporter 1 (vGluT1; red) immunostaining show overlap (yellow), indicating that ATRAP is localized in synaptic terminals of photoreceptors. (**c**) ATRAP immunostaining (green) overlaps with immunostaining for CtBP2 (red), a synaptic ribbon marker. Images on the right (**a’–c’**) are higher magnifications of retinal regions delimited by the boxes shown in (**a**–**c**). Outer nuclear layer (ONL), outer plexiform layer (OPL), inner nuclear layer (INL), inner plexiform layer (IPL), ganglion cell layer (GCL). (**d)** Bar graph showing the thresholded Pearson’s correlation coefficient values for colocalization of ATRAP immunostaining with vGluT1, CtBP2, and GFP-Rac. Large values indicate stronger colocalization, values close to 0 lack of colocalization. Data are mean ± SEM, n = 4 in each group.

The finding that ATRAP is expressed in the OPL (Fig. 1) but not in a variety of second-order neurons (Fig. 3) leaves photoreceptor cells or OFF cone bipolar cells as likely candidates for ATRAP expression. We first use a transgenic mouse line (Rac3-eGFP) in cone photoreceptor cells express enhanced green fluorescence protein (EGFP) to test whether these cells also express ATRAP (Fig. 4a,d). We find robust co-localization of ATRAP with EGFP in the cone photoreceptor cells photoreceptor cells pedicles (Fig. 4a) and some ATRAP labeling beyond the boundaries of the cone photoreceptor terminals, which presumably corresponds to rod photoreceptor terminals (Fig. 4a). Next, we explore the subcellular localization of ATRAP in photoreceptors. ATRAP immunostaining localizes to synaptic terminals identified with the vesicular glutamate transporter 1 (vGluT1)^34,35^; (Fig. 4b,d). Furthermore, double-labeling sections of mouse retina with antibodies against ATRAP and C-terminal binding protein 2 (CtBP2), a transcriptional corepressor and specific marker for photoreceptor ribbon structures^36^, shows a strong co-localization of both proteins at photoreceptor terminals (Fig. 4c,d). These results demonstrate that ATRAP is likely expressed at the presynaptic side of both rod and cone photoreceptor terminals.

**Figure 5:**
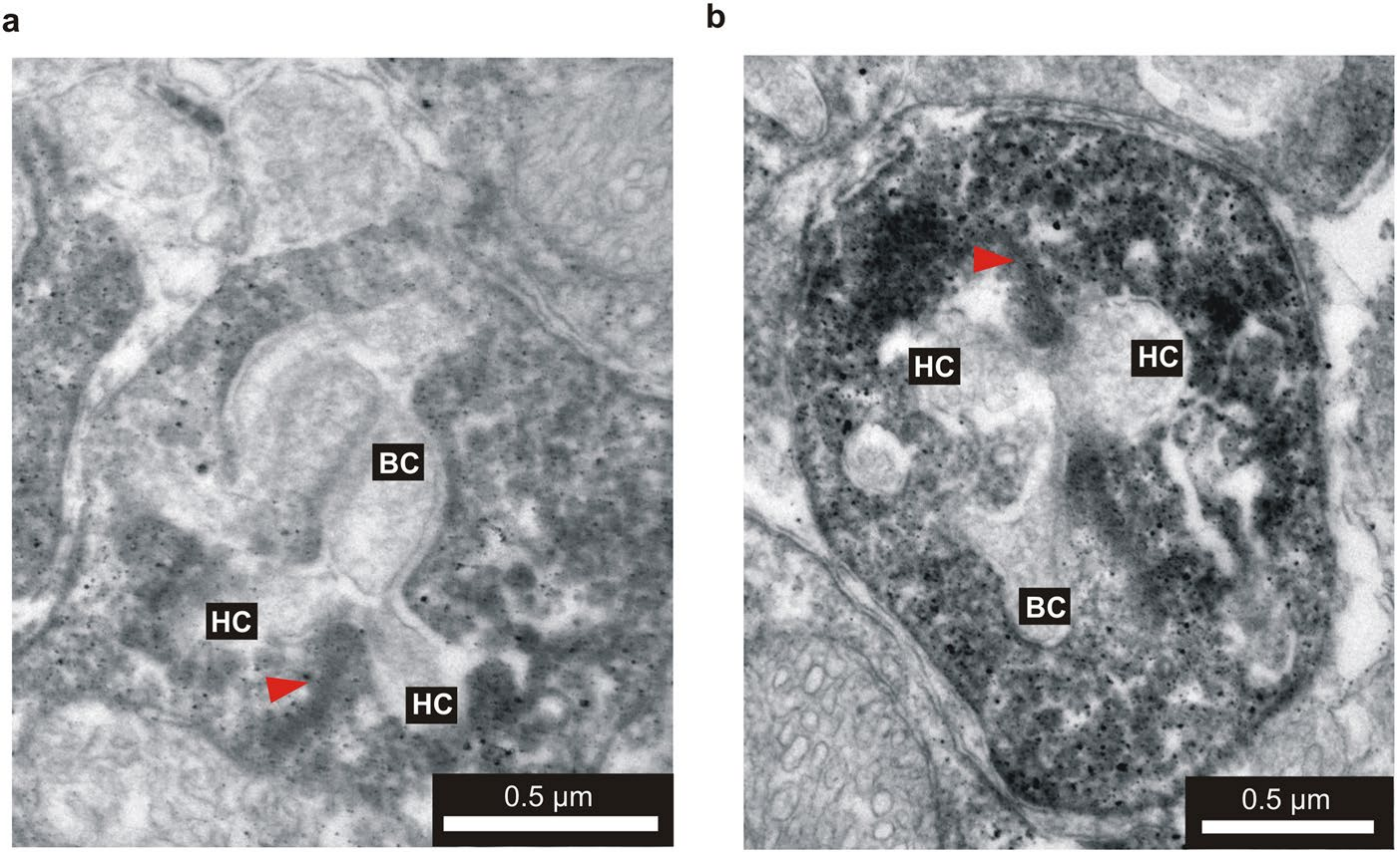
Ultrastructural localization of ATRAP by pre-embedding immunoelectron microscopy. High-power electron micrographs showing the ultrastructural localization of ATRAP immunoreactivity in the OPL. (**a,b**) ATRAP staining in the OPL was confined to photoreceptor presynaptic terminals; as indicated by the presence of a synaptic ribbon (red arrowheads). Lateral horizontal cell (HC) and central bipolar cell dendrites (BC) are devoid of immunogold particles.

We further analyze the ultrastructural distribution of ATRAP in photoreceptor terminals by means of immunogold labeling in transmission electron microscopy. ATRAP immunoreactivity localizes to membranous structures inside photoreceptor terminals; the postsynaptic dendrites of bipolar cells and horizontal cells show no ATRAP labeling (Fig. 5).

### ATRAP modulates Ca^2+^ signals in the presynaptic terminals of photoreceptors

**Figure 6:**
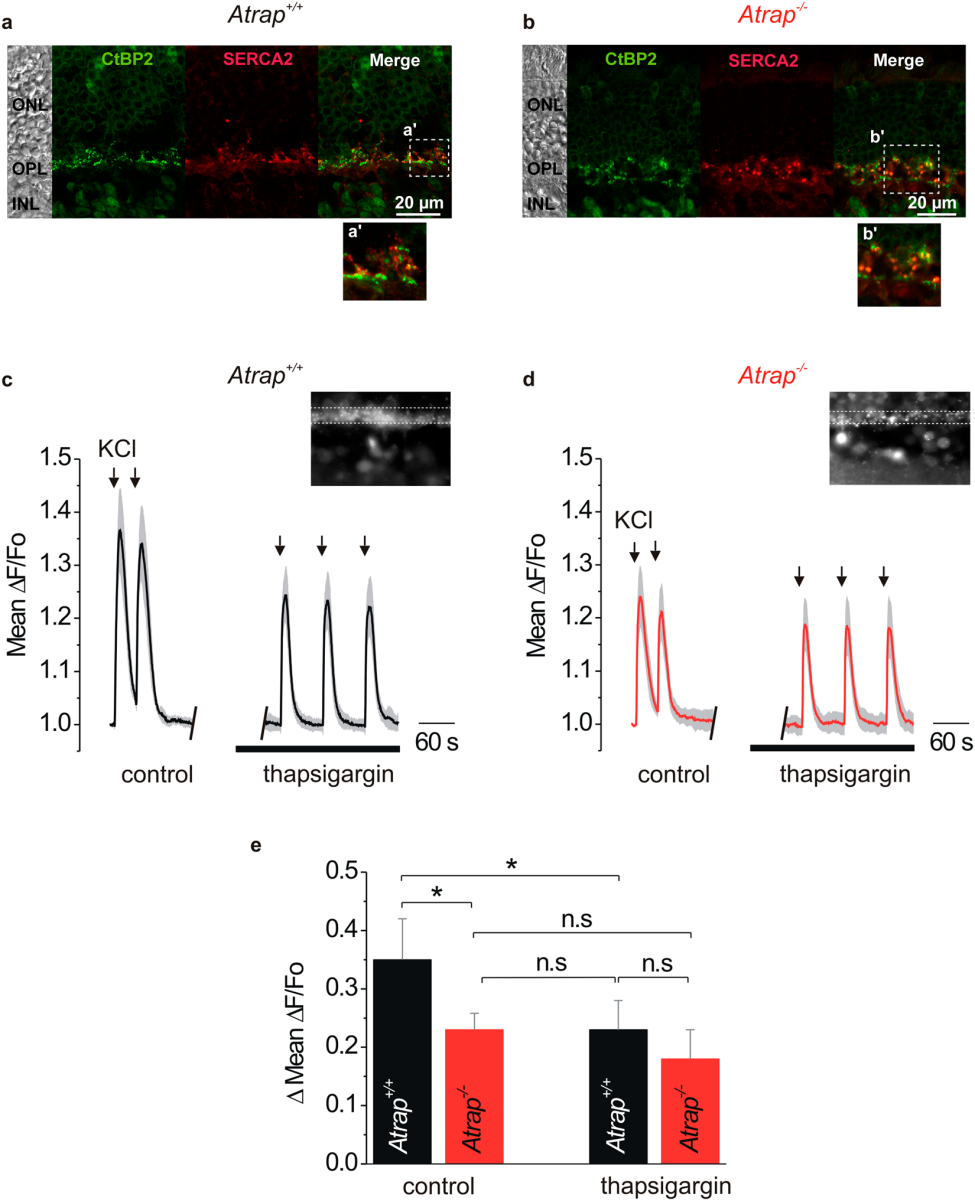
ATRAP modulates Ca^2+^ signals in the presynaptic terminals of mouse photoreceptors. **(a,b**) Confocal images of vertical sections from *Atrap*^+/+^ (**a**) and *Atrap*^*−/−*^ (**b**) mouse retinae show that CtBP2 (green) and SERCA2 immunostaining (red) overlap (yellow). (**a’**) and (**b’**) are higher magnifications of retinal regions delimited by the boxes shown in (**a**,**b**). (**c,d**) Local application of KCl (150 mM, 3 s; arrows) evokes depolarization-induced Ca^2+^ transients in photoreceptor terminals in vertical slices from *Atrap*^+/+^ (**c**) and *Atrap*^*−/−*^ mouse retinae (**d**). Traces show the mean (black) ± SEM (grey) of 6 retinae in each group. Lines under the traces represent application of the SERCA2 blocker thapsigargin (1 µM). Insets show Fluo-4 loaded photoreceptor terminals. (**e)** Quantification of data shown in (**c**) and (**d**). Data are mean ± SEM of the peak amplitudes (ΔMean ΔF/F_0_), n = 10. * indicate significant difference (p < 0.05, ANOVA).

It has been shown previously that retinal pigment epithelial (RPE) cells and cardiac myocytes from *Atrap*^*−/−*^ mice have altered store-mediated Ca^2+^ responses compared to *Atrap*^*+/+*^ mice; in cardiac myocytes, ATRAP functions as an activator of SERCA^6,7^. We thus investigate how ATRAP contributes to the dynamics of intracellular Ca^2+^ in photoreceptor terminals, focusing on the contribution of intracellular Ca^2+^ stores to depolarization-induced Ca^2+^ responses. Since ATRAP enhances the activity of SERCA2 by physical interaction and SERCA2 has been identified as the major Ca^2+^-ATPase pump in the photoreceptor synapse^25^, we examine the expression of SERCA2 by means of immunohistochemistry in *Atrap*^*+/+*^ and *Atrap*^*−/−*^ mice. SERCA2 is expressed in retinal sections from both *Atrap*^*+/+*^ and *Atrap*^*−/−*^ mice (Fig. 6a,b). SERCA2 immunostaining is strongest in the OPL, where it co-localizes with the ribbon marker CtBP2 (Fig. 6a,b). Thus, presynaptic terminals of photoreceptors abundantly express both SERCA2 and ATRAP.

**Figure 7:**
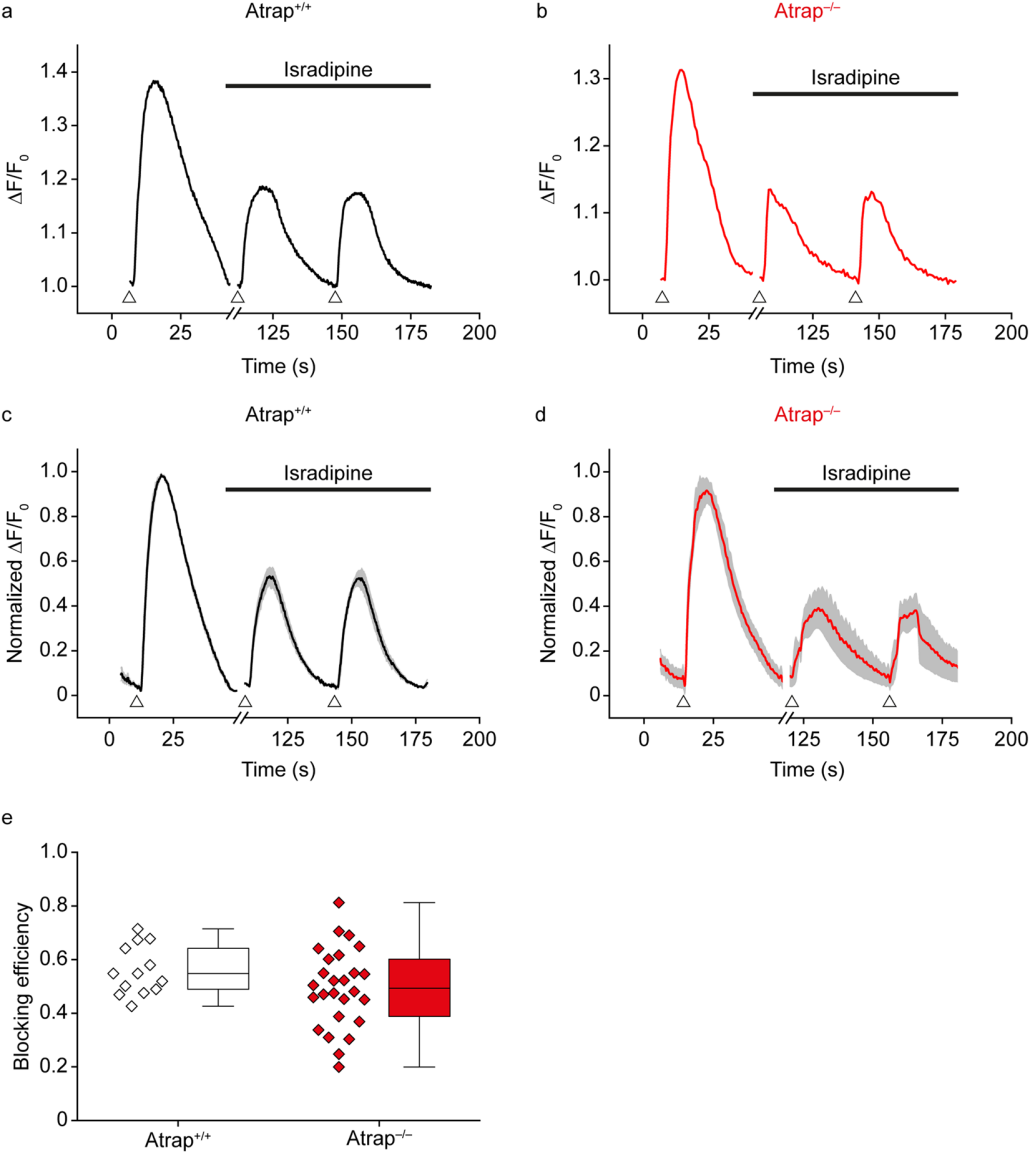
Isradipine blocks depolarization-evoked Ca^2+^ signals in the photoreceptor synaptic layer of the retina. (**a,b**) Representative Ca^2+^ responses of photoreceptor terminals of *Atrap*^*+/+*^ (**a**) and *Atrap*^*–/–*^ mice (**b**) to extracellular application of 150 mmol/l KCl in the presence of 10 µmol/l isradipine. Arrowheads indicate application of KCl (3 s duration). (**c,d**) Average responses of all photoreceptor terminals of *Atrap*^*+/+*^ (n = 3; **c**) and *Atrap*^*–/–*^ mice (n = 5; **d**). Each trace was normalized to its maximum amplitude in the absence of isradipine. Grey areas indicate s.e.m. (**e)** Box plot summarizing the blocking efficiency of isradipine. Each data point corresponds to a region of interest (animal numbers as given above). For all experiments, regions of interest were defined as rectangles (10 × 15 µm) covering photoreceptor terminals in the OPL.

To test whether ATRAP has an effect on Ca^2+^ signaling at photoreceptor terminals, we perform Ca^2+^-imaging experiments on vertical slices of acutely isolated retinae from both *Atrap*^*+/+*^ and *Atrap*^*−/−*^ mice. Since our experimental setup does not allow to combine imaging with light responses, we simulate the switch from light to dark with a depolarizing stimulus. We repeatedly expose the retinae to extracellular KCl (150 mM, 3 s) depolarize the membrane of photoreceptors to approximately 0 mV, causing the opening of voltage-gated Ca^2+^ channels. We tested whether the dihydropyridine Ca^2+^ channel blocker isradipine had a differential effect on Ca^2+^ influx into photoreceptor terminals of *Atrap*^*+/+*^ and *Atrap*^*–/–*^ animals. Slices were incubated with the Ca^2+^ indicator Fluo4-AM and subjected to extracellular application of 150 mmol/l KCl. KCl-depolarization of *Atrap*^*–/–*^ retinal sections induces ^2+^ responses that are significantly smaller than in *Atrap*^*+/+*^ retinal sections (Fig. 6c–e). Application of thapsigargin (1 µM), an inhibitor of SERCA2, significantly reduces the depolarization-induced ^2+^-peaks in retinae of *Atrap*^*+/+*^ mice ((Fig. 6c,e), indicating that the release from intracellular stores contributes to the Ca^2+^ signal at the presynaptic terminal. In retinae of *Atrap*^*–/–*^ mice, by contrast, thapsigargin treatment has no significant effect on the amplitude of depolarization-induced Ca^2+^ peaks (Fig. 6d,e). Thus, compared to *Atrap*^*+/+*^ mice, the depolarization-evoked Ca^2+^ signals in the photoreceptor presynaptic terminals of *Atrap*^*–/–*^ mice are smaller and insensitive to thapsigargin inhibition. Figure 7a shows the response recorded from a single region of interest (ROI) from a wildtype mouse retina. Due to its size, the ROI encompassed several photoreceptor terminals. Cells were depolarized twice in the presence of isradipine (10 µmol/l) to allow for complete block of voltage-gated Ca^2+^ channels. The control response was reduced to about 50% by isradipine. Since we observed no differences of Ca^2+^ amplitudes between the two KCl applications in the presence of isradipine, the arithmetic mean was used for further data analysis. In photoreceptors of *Atrap*^*–/–*^ mice, isradipine had a very similar effect on the KCl-induced Ca^2+^ response (Fig. 7b). This observation was confirmed when all measurements were averaged and normalized (Fig. 7c,d). The ratios of peak amplitudes in the absence and presence of isradipine, showed no significant difference (Fig. 7e). Mean values were 0.559 ± 0.026 (13 ROIs from 3 animals) in *Atrap*^*+/+*^ and 0.494 ± 0.029 (26 ROIs from 5 animals) in *Atrap*^*–/–*^ mice.

**Figure 8:**
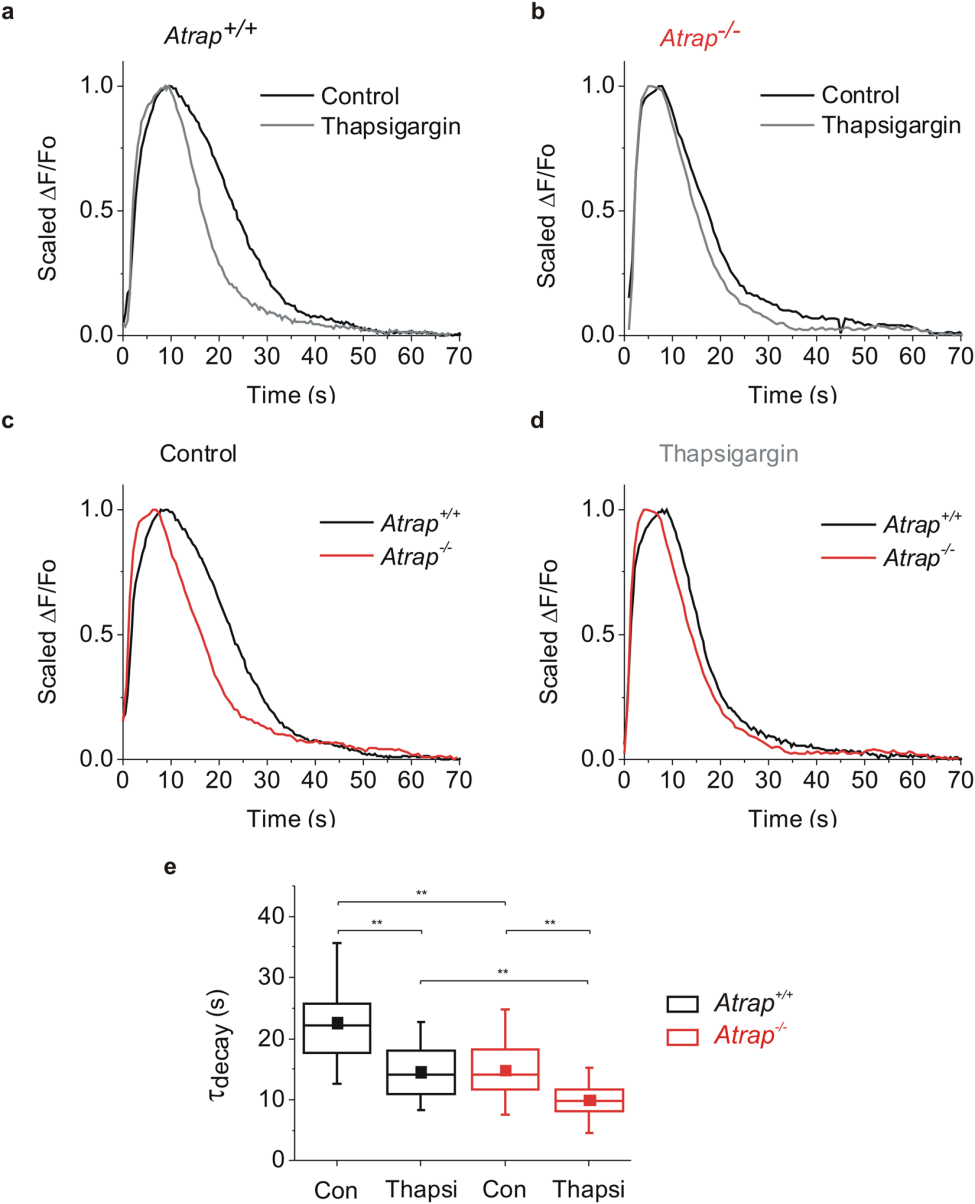
ATRAP influences the time course of depolarization-evoked Ca^2+^ signals in photoreceptor terminals. (**a,b**) KCl (150 mM, 3 s) depolarization-evoked Ca^2+^ signals in slices from *Atrap*^+/+^ (**a**) and *Atrap*^*−/−*^ (**b**) mouse retinae before (black) and after application of thapsigargin (1 µM, gray). (**c,d**) KCl (150 mM, 3 s) depolarization-evoked Ca^2+^ signals in slices from *Atrap*^+/+^ (black) and *Atrap*^*−/−*^ mouse retinae (red) under control conditions (**c**) and in the presence of thapsigargin (**d**). Traces of Ca^2+^ signals were scaled to peak amplitudes. (**e**) Quantification of time constant decays of data shown in (a-d). n = 10, ** indicate significant difference (p < 0.01, ANOVA).

To get further insight into the functional role of ATRAP, we analyze temporal aspects of individual depolarization-induced Ca^2+^ signals in photoreceptor terminals (Fig. 8). Inhibiting SERCA2 with thapsigargin leads to a faster decay of the Ca^2+^ signal in retinal slices from both *Atrap*^*+/+*^ and *Atrap*^*−/−*^ mice compared to their respective controls, indicating that SERCA2 activity modulates the time course of Ca^2+^ responses (Fig. 8a,b,e). Furthermore, the decay of the Ca^2+^ responses in retinal slices from *Atrap*^*−/−*^ mice is faster than in retinal slices from *Atrap*^*+/+*^ mice (Fig. 8c,e). Interestingly, the decay of Ca^2+^ signals in retinal slices from *Atrap*^*−/−*^ mice under control conditions has the same time constant as that of Ca^2+^ signals in retinal slices from *Atrap*^*+/+*^ mice in the presence of thapsigargin (Fig. 8d,e). These results indicate that the lack of ATRAP alters the Ca^2+^ signaling in the presynaptic terminal in a manner that closely resembles the effects of blocking SERCA2.

**Figure 9:**
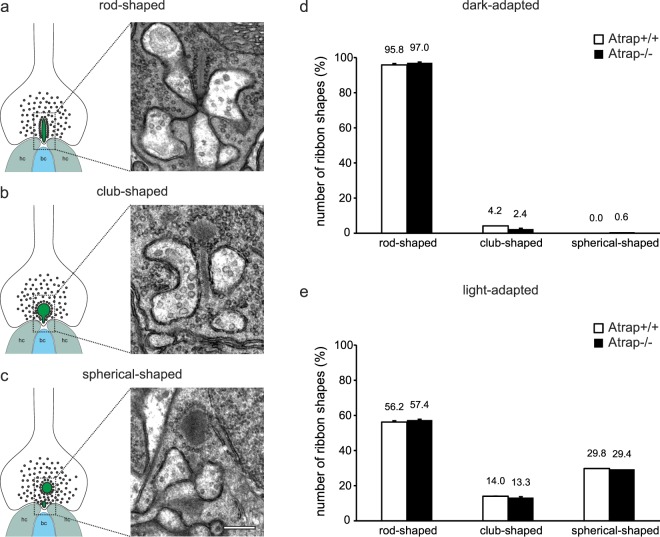
Ultrastructural analysis of photoreceptor synaptic ribbons in dark- and light-adapted *Atrap*^*+/+*^ and *Atrap*^*−/−*^ mice. (**a–c**) Schematic drawings and representative electron micrographs of (**a**) rod-shaped, (**b**) club-shaped, and (**c**) spherical-shaped SRs. (**d-e**) Number of SR shapes in dark- and light-adapted *Atrap*^*+/+*^ and *Atrap*^*−/−*^ mice in percent. Error bars are shown as SD. n = 2 animals per genotype and illumination condition. Scale bar = 0.2 µm for (**a–c**).

Recent work showed that Ca^2+^ mediates the changes in the morphology of the photoreceptor synaptic ribbons between light and dark adaptation^37,38^. To investigate whether the morphology of photoreceptor synaptic ribbons (SRs) between light and dark conditions is affected in *Atrap*^*−/−*^ mice, we compare the shape of SRs between *Atrap*^*+/+*^ and *Atrap*^*−/−*^ mice following dark or light adaptation according to Fuchs *et al*.^39^. We examine several hundred electron micrographs for each genotype and illumination condition and classified SRs according to their shape into three different categories:rod-shaped, club-shaped, and spherical-shaped (Fig. 9a–c). In dark-adapted *Atrap*^*+/+*^ mice, the majority of SRs was rod-shaped (95.8% of 320 SRs), while the remaining SRs appeared club-shaped (4.2% of 320 SRs). Similar results were obtained for *Atrap*^*−/−*^ mice, with the majority of SRs being rod-shaped (97.0% of 346 SRs) and only very few club-shaped (2.4% of 346 SRs) and spherical-shaped SRs (0.6% of 346 SRs) (Fig. 9d). Light adaptation caused in both genotypes a comparable reduction in the number of rod-shaped SRs (*Atrap*^*+/+*^: 56.2% of 362 SRs; *Atrap*^*−/−*^: 57.4% of 443 SRs) and an increase in the number of club-shaped (*Atrap*^*+/+*^: 14% of 362 SRs; *Atrap*^*−/−*^: 13.3% of 443 SRs) and spherical-shaped SRs (*Atrap*^*+/+*^: 29.8% of 362 SRs; *Atrap*^*−/−*^: 29.4% of 443 SRs) (Fig. 9e).

### ATRAP modulates Ca^2+^ signaling at the mossy fiber terminals in the cerebellum

**Figure 10:**
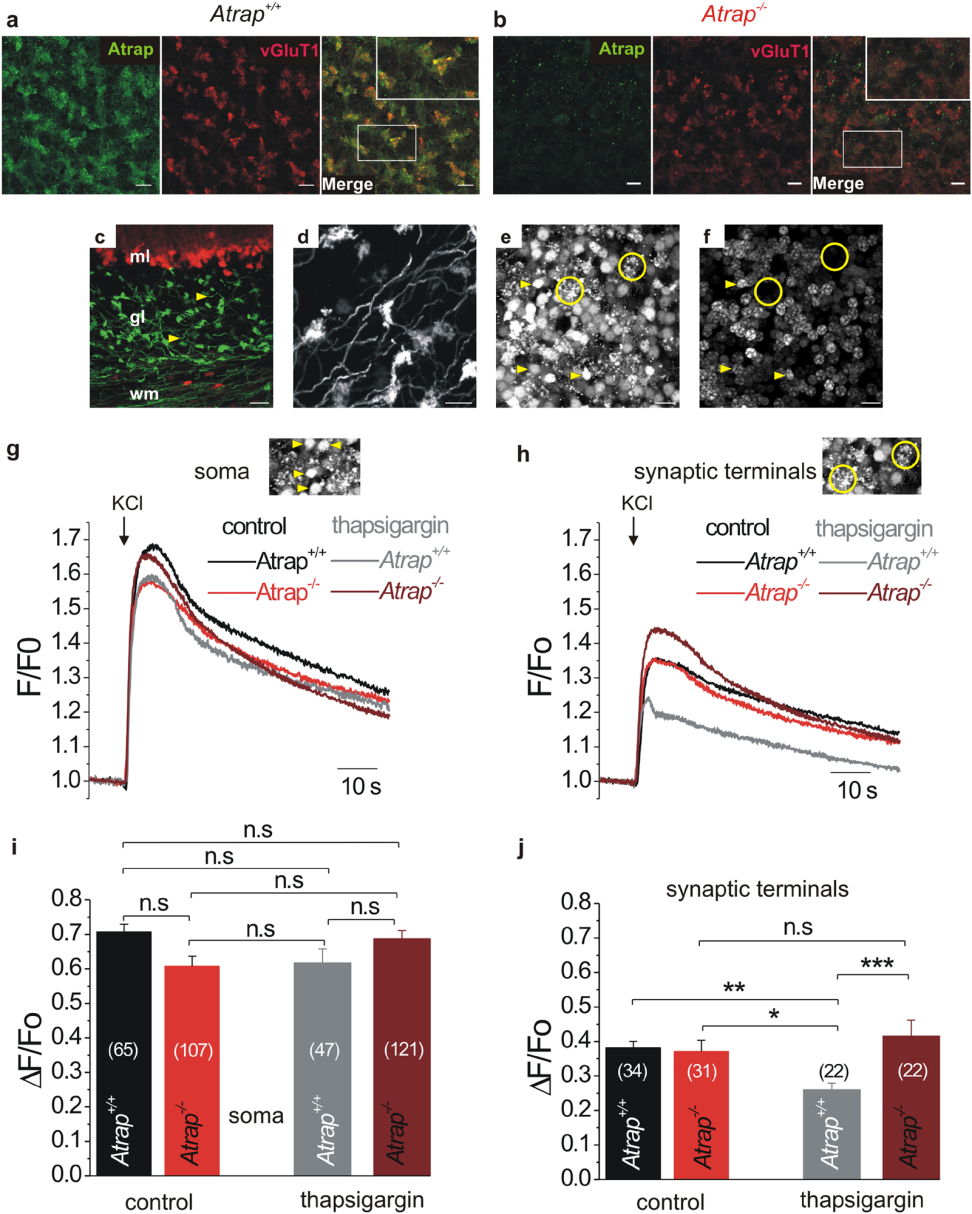
ATRAP modulates Ca^2+^ signals in presynaptic mossy fiber terminals of the mouse cerebellum. (**a,b**) Confocal images of sagittal sections from *Atrap*^+/+^ (**a**) and *Atrap*^*−/−*^ (**b**) mouse cerebellum. Double labeling for ATRAP (green) and vGluT1 (red) showed overlap (yellow), indicating that ATRAP colocalized to presynaptic terminals (**a**). Note that ATRAP staining was absent in sections from *Atrap*^*−/−*^ mouse cerebellum (**b**). Insets show higher magnifications of cerebellar regions delimited by the boxes. (**c**) Confocal images of the cerebellum of a GFAP-ECFP/Thy1-EYFP transgenic mouse. Yellow arrowheads pointing to EYFP-labeled mossy fiber terminals (green). ECFP-expressing Bergmann glia cells are shown in red. Molecular layer (ml), granular layer (gl), white matter (wm). (**d**) A magnified view from (**c**) reveals the glomerular synaptic network structure of mossy fiber terminals. (**e,f**) Confocal images of parasagittal sections from *Atrap*^+/+^ mouse cerebellum. In (**e**) the labelling with Fluo-4 AM and in (**f**) the nuclear staining of the same view with DRAQ5, a red fluorescent DNA dye. Note that in (**e**) the Fluo-4-labeled presynaptic terminal structures (yellow circles) are readily distinguished from labeled granule cell nuclei (yellow arrowheads). (**g,h**) Local application of KCl (150 mM, 5 s; arrows) evokes depolarization-induced Ca^2+^ transients in (**g**) Fluo4-labeled mossy fiber soma or (**h**) mossy fiber synaptic terminals from *Atrap*^+/+^ (black and gray) and *Atrap*^*−/−*^ (red and wine) mouse cerebellum with or without thapsigargin (5 µM). Note the significant reduction of Ca^2+^ response amplitudes in thapsigargin-treated terminals of *Atrap*^+/+^ mice that is absent in *Atrap*^*−/−*^ mice (**h**). Insets show Fluo-4-labeled granule cell nuclei (yellow arrowheads) and Fluo-4-labeled presynaptic terminal structures (yellow circles) as in (**e**). **(i,j**) Quantification of data shown in (**g**,**h**) respectively. Data are mean ± SEM of the peak amplitudes (ΔF/F_0_), (numbers of analyzed terminals are given within the bars). Tukey’ post-hoc ANOVA test was used for statistical analysis. Scale bars = 10 µm.

The photoreceptor synapse is specialized for the continuous release of glutamate. As ATRAP mRNA is also found in the brain^4^, it might be possible that ATRAP has a more generalized function at chemical synapses of the brain. We find strong ATRAP immunolabeling in the cerebral cortex and the cerebellum of *Atrap*^*+/+*^ mice. (Fig. 2). The cerebellum is a highly organized and stratified brain area that facilitates the analysis of synaptic structure and function^40^. Thus, we focused the next step of our investigation onto the cerebellum to prove the hypothesis that the synaptic ATRAP function does not only apply for photoreceptors. Strong immunoreactivity of ATRAP colocalize with the presynaptic glutamate transporter 1 (vGluT1) in the glomeruli formed by mossy fiber terminals and granule cell dendrites (Fig. 10a) and is absent in *Atrap*^*−/−*^ mice (Fig. 10b). In GFAP-ECFP/Thy1-EYFP double-transgenic mice, the mossy fiber terminals can be readily identified in the granule cell layer, while fluorescently tagged Bergmann glial cells outline the Purkinje cell layer below the molecular layer (Fig. 10c,d). Since the specific localization of ATRAP at presynaptic terminals of the cerebellum suggests a similar role as in the retina, we test this hypothesis by Ca^2+^-imaging on acute cerebellar slices isolated from both *Atrap*^*+/+*^ and *Atrap*^*−/−*^ mice. We discriminate cell somata from mossy fiber synaptic terminals by the unique morphology of Fluo4-AM fluorescent glomerular structures (yellow circles in Fig. 10e). Control labeling of cell nuclei by DRAQ5 reveals a clear distinction between synaptic and somatic Fluo4-AM loading (Fig. 10e,f). Local application of 150 mM KCl via a patch-pipette is used to activate voltage-gated Ca^2+^ channels in cerebellar slices for 5 s. As shown in Fig. 10g,i, KCl-depolarization induces similar Ca^2+^ responses in the somata of granule cells in both *Atrap*^*+/+*^ and *Atrap*^*−/−*^ mouse cerebellum in the presence or absence of thapsigargin, as one would expect for a neuronal compartment in which plasma membrane-localized voltage-gated Ca^2+^ channels predominate. In the glomerular structures containing the presynaptic terminals of mossy fibers, however, depolarization-evoked Ca^2+^ responses are also mediated by Ca^2+^ release from intracellular stores, since inhibition of SERCA by thapsigargin significantly reduces the response amplitude (Fig. 10h,j). By contrast, in the synaptic terminals of *Atrap*^*−/−*^ mice, application of thapsigargin does not change the Ca^2+^ response (Fig. 10h,j red vs maroon). Together, these results suggest that, just as in photoreceptors, ATRAP is involved in store-mediated Ca^2+^ signaling at the synaptic terminal of mossy fibers in the cerebellum.

## Discussion

While studying the functional role of ATRAP in the retina to further understand the physiological role of the RAS in the retina, we found that ATRAP shapes synaptic Ca^2+^ transients through mechanisms not involving classical AT1R signaling. Here we present molecular, structural and physiological evidence for a novel function of ATRAP as a synaptic protein within the retina and the cerebellum that participates in the generation of Ca^2+^ signals. ATRAP appears to function by modulating the Ca^2+^ release from intracellular stores, as we find in selected synapses of brain regions as diverse as cerebellum and retina. Therefore, we hypothesize that ATRAP may contribute more broadly to Ca^2+^ signaling in neurons, rather than in its conventional role in the angiotensin receptor signaling.

ATRAP is expressed in a variety of organs including the heart, aorta, lung, adrenal glands, liver, spleen, testis, and brain, and is most abundant in the kidney^4^. ATRAP interacts with the cytosolic side of AT1R^4^. Although the classical function attributed to ATRAP is to modulate angiotensin-receptor signaling, we recently showed that ATRAP is also important in Ca^2+^ store-dependent Ca^2+^ signaling in the RPE^6^. Moreover, in cardiac myocytes ATRAP physically interacts and stimulates SERCA2 activity^7^. ATRAP further binds to proteins not related to angiotensin II signaling, such as Ca2^+^ modulating cyclophilin ligand and receptor for activated C kinase-1^8,41^. Along these lines, ATRAP has also been associated with retinal degradation type B protein, a member of the phosphatidyl inositol transfer protein family that functions in retrograde transport from the Golgi to the endoplasmic reticulum^42^. In the retina, we found ATRAP predominantly expressed in the RPE and the OPL. However, as previously shown^30^, we find no expression of the AngII receptor in the OPL (Fig. 1D) when using an antibody against AT1R that shows no false positive staining in the AT1R knockout mouse kidney. Because the OPL lacks expression of AngII-receptor and because ATRAP mainly localizes to intracellular structures distant from the plasma membrane, we postulate that ATRAP is not involved in the conventional AngII-signaling pathway in the mammalian retina. Instead, ATRAP’s localization suggests a role in Ca^2+^ signaling in photoreceptors presynaptic terminals.

We gain insight into the role of ATRAP in photoreceptor terminals by performing Ca^2+^ imaging in the OPL in acute retinal slices from both *Atrap*^*+/+*^ and *Atrap*^*−/−*^ mice. We focus on intracellular Ca^2+^ stores because: 1) the lack of ATRAP in RPL cells or in cardiac myocytes reduces the store-mediated Ca^2+^ response upon extracellular stimulation^6,7^ and 2) Ca^2+^ release from intracellular stores contributes to the depolarization-induced Ca^2+^ signaling at photoreceptor presynaptic terminals that corresponds with a transition from light to darkness^20,21,25^. To mimic this transition, we used pulses of extracellular high K^+^ concentrations that lead to photoreceptor cell depolarization. In the presence of the L-type Ca^2+^ channel blocker isradipine, we observed a similar reduction of the Ca^2+^ transients (by 85–90%) in *Atrap*^*−/−*^ and *Atrap*^*+/+*^ mice. This result indicates that the level of Ca^2+^ signals were predominantly measured in photoreceptor terminals and that the level of depolarization together with number of activated L-type channels were equal in both *Atrap*^*−/−*^ and *Atrap*^*+/+*^ mice. Photoreceptor terminals in retinae from *Atrap*^*−/−*^ mice show a smaller and thus faster recovery of depolarization-evoked Ca^2+^ transients. It was shown^37,38^ that the shape of the synaptic ribbon changes between light and dark adaptation through a Ca^2+^ dependent mechanism. Using electron microscopy techniques, we here showed that the shape of the photoreceptor synaptic ribbons from *Atrap*^*+/+*^ and *Atrap*^*−/−*^ mice under light and dark adaptation are similar. Thus, the absence of ATRAP has no influence on the synaptic ribbon, indicating that the function is not influenced as well. In addition, depolarization-evoked Ca^2+^ transients in retinae from *Atrap*^*−/−*^ mice are insensitive to thapsigargin and have similar decay times to that of *Atrap*^*+/+*^ retinae in the presence of the SERCA blocker thapsigargin (Fig. 6E), suggesting that ATRAP is required to activate the SERCA pump during presynaptic Ca^2+^ signaling in photoreceptors. The absence of ATRAP has similar effects than SERCA inhibition. Inhibition of SERCA results in a reduced concentration of Ca^2+^ in cytosolic Ca^2+^ stores because Ca^2+^ constantly leaks out of the stores and is not pumped back. Therefore, SERCA inhibition alone suffices to induce store-operated Ca^2+^ entry in many different cell types^43^. The smaller amplitude of depolarization-evoked Ca^2+^ transients results from reduced amounts of Ca^2+^ released from stores in the *Atrap*^*−/−*^ mouse photoreceptors. Thus, ATRAP likely has a similar molecular function in photoreceptor synapses than in cardiac myocytes^7^, however, differing by the fact that in photoreceptors ATRAP does not need AT1R receptor activation.

Our data indicate that ATRAP is involved in Ca^2+^ store-dependent signaling in the photoreptor synapse. Therefore we speculate that the physiological role of ATRAP in the photoreceptor synapse is linked to regulate the Ca^2+^ store signaling of the light-dependent increase in glutamate release. Transition from light to dark depolarizes photoreceptors, which activates L-type Ca^2+^ channels and stimulates Ca^2+^ release from endoplasmic Ca^2+^ stores via Ca^2+^ dependent activation of ryanodine receptors^9^. Ca^2+^-release in turn activates store-operated Ca^2+^ entry^44^. This additional store-operated Ca^2+^ entry further increases the Ca^2+^ transient to strengthen and amplify the signal^20,44^. Importantly, the store-operated Ca^2+^ entry boosts vesicular glutamate release at the photoreceptor synapse in general^14,16,21,45^. ATRAP activates SERCA2, which in turn pumps Ca^2+^ into cytosolic Ca^2+^ stores, that implying that ATRAP supports glutamate release by maintaining high levels of Ca^2+^ inside the stores. Finally, because the Ca^2+^ signals from *Atrap*^*−/−*^ photoreceptors were insensitive to thapsigargin, it is likely that the boost effect does not occure in the absence of ATRAP.

Since the expression of ATRAP is not restricted to the retina and can be found in the cortex and in the cerebellum, we analyzed KCl-evoked Ca^2+^ transients in presynaptic structures of cerebellar mossy fibers. Early studies already showed that Ca^2+^ release from stores plays a role in generating large miniature inhibitory postsynaptic currents (IPSCs) in cerebellar Purkinje cells^17–19^. Here, the generation of large-amplitude miniature IPSCs in cerebellar Purkinje cells depends on activation of ryanodine receptors at the presynapse of interneurons^17,18^. In our experiments, inhibition of SERCA by thapsigargin reduces depolarization-evoked presynaptic Ca^2+^ signals in mossy fibers, further implying a contribution of Ca^2+^ release from stores. However, in contrast to wildtype mice, the Ca^2+^ transients in mossy fibers of *Atrap*^*−/−*^ mice are insensitive to thapsigargin. This result equals to that in photoreceptor synapses from *Atrap*^*−/−*^mice and it is tempting to speculate that ATRAP fulfills a related role in Ca^2+^ signalling in cerebellar synapses. Moreover, because the cerebellum, unlike the photoreceptors, shows a broad AT1R expression^46^, the function of ATRAP in mossy fibers synapses could also suggest the existence of novel mechanisms of the renin-angiotensin system network in the brain.

In contrast to photoreceptor terminals, the depolarization-evoked Ca^2+^ transient amplitude is unchanged in cerebellar mossy fiber cells from *Atrap*^*−/−*^ mice. Furthermore, cardiac myocytes from *Atrap*^*−/−*^ mice also show unchanged Ca^2+^ amplitudes in response to depolarization compared to those from wild-type mice^7^. Thus, the lack of ATRAP has very different effects on depolarization-evoked Ca^2+^ transients in photoreceptors, mossy fibers and cardiac myocytes. This suggests that, depending on the cell type, ATRAP-dependent modulation of SERCA2 activity affects in different ways the dynamics of Ca^2+^ signaling.

To date, ATRAP function was assumed to be limited to modulating the AT1R-mediated angiotensin II signaling cascade. Our study shows that, in addition to its conventional role in angiotensin signaling, ATRAP may be also crucial for the Ca^2+^ signaling in synapses of the retina and the cerebellum. Since ATRAP expression localizes to different brain areas it is possible that this synaptic ATRAP function is of more broad relevance.

## Methods

### Animals and ethical approval

Animals and ethical approval followed the conditions as published previously^6^. Adult male and female ATRAP knockout (*Atrap*^*−/−*^) mice (C57BL/6 × 129SvEv) from a local colony were used in the study^4^. *Atrap*^*−/−*^ and *Atrap*^+/+^ littermates were used from heterozygous breeding pairs. All experimental procedures were performed following the guidelines approved by the Institutional Animal Care and Use Committee at University of Regensburg and the Association for Research in Vision and Ophthalmology (ARVO) statement for the use of animals in vision research. All animal experiments were formally approved by the German authorities (Bavarian Administration/Regierung Oberpfalz under number: 54-2532.1-06/10).

GFAP-ECFP/Thy1-EYFP transgenic mice were bred in the animal facility of the University of Saarland in Homburg and used for confocal imaging of the cerebellum after perfusion fixation as previously described^47^.

Tg(Rac3-EGFP)JZ58Gsat/Mmcd (Rac3-EGFP) mice were obtained from the Mutant Mouse Regional Resource Center, a NCRR-NIH funded strain repository, and were donated to the MMRRC by the NINDS funded GENSAT BAC transgenic project.

### Laser capture microdissection

For laser capture microdissection, unfixed eyes were prepared, frozen and sectioned at a thickness of 25 µm using a cryostat. Defined regions from the outer and inner plexiform layers were dissected and collected on the same day using the PALM Micro Beam system (Carl Zeiss MicroImaging) equipped with a nitrogen laser (337 nm). After microdissection, the samples were ejected from the object plane with a single laser shot and catapulted directly into a microtube cap (Adhesive-Cap, Carl Zeiss MicroImaging) for subsequent reverse transcriptase-PCR.

### Reverse transcriptase-PCR (RT-PCR) analysis

RT-PCR was performed using the method as described by Mühlhans *et al*.^48^. Freshly micro-dissected tissue was homogenized in RLT buffer (Qiagen, Hilden, Germany) containing 1% β-mercaptoethanol. RNA was isolated using the RNeasy Micro Kit (Qiagen). Poly(dT)- and random hexamer-primed cDNA synthesis (reverse transcriptase reaction) was performed for 30 min at 42 °C using 5x RT-buffer, a mixture of dNTPs, RNAsin (all from Promega, Mannheim, Germany) and complete RNA from microdissected tissue. PCR was performed in a volume of 25 µl using 3 µl of prepared cDNA and 0.5 µl of each primer. Cycling conditions were 40 cycles at 95 °C for 45 s, 42–65 °C for 45 s, and 72 °C for 1 min followed by a 5-min 72 °C extension step. PCR product lengths were determined on 1.5–2% agarose gels. Primers specific for mouse ATRAP taken from^49^ were as follows:

Primer ATRAP forward: 5′-TGCTTGGGGCAACTTCACTATC-3′

Primer ATRAP reverse: 5′-ACGGTGCATGTGGTAGACGAG-3′

Primers to opsin and β-actin used as controls were as follows:

Primer opsin forward: 5′-CTCTTCTGCATCTTCTCT-3′

Primer opsin reverse: 5′-AGGGTTTACAGATGACAA-3′

Primer β-actin forward: 5′-TTCCTCCCTGGAGAAGAG-3′

Primer β-actin reverse: 5′-CACTGTGTTGGCATAGAG-3′

### Immunohistochemistry

After dissection, retinas and brain slices were fixed in 4% paraformaldehyde in 0.1 M phosphate buffer (PB) for 25 min and washed in PB for 30 min. The tissue was frozen and sectioned at a thickness of 12 µm using a cryostat. Sections were incubated in a solution containing 5% normal goat serum (NGS) and 0.3% Triton X-100 in PB for 1 h, and they were subsequently transferred for an overnight incubation to a solution containing primary antibodies. The following primary antibodies were used in separate experiments: rabbit anti-ATRAP (1:1,000; Davids Biotechnologie, Regensburg, see^4^, rabbit anti ANG II receptor (1:1,000, ab47408; Abcam, Cambridge, UK), rabbit anti-calbindin (1:1,000) and mouse anti-calbindin (1:1,000; both Swant, Marly, Switzerland), mouse anti-CtBP2 (1:20,000; BD Transduction, Heidelberg, Germany), G_o_α (1:10,000; Merck Millipore, Billerica, MA), vGluT1 (1:50,000; Merck Millipore, Billerica, MA), anti-SERCA2 (1:200; Pierce MA3–910/IID8, Pierce Bonn). Since it known that anti-AT1R antibodies often lack reliability we checked every batch of antibodies by staining of kidney sections from AT1R/AT2R double knock out mice e.g. see Milenkovic *et al*.^30^. Secondary antibodies conjugated to Alexa Fluor 488 and Alexa Fluor 594 (both Molecular Probes, Eugene, OR, USA) were dissolved in 1% NGS and 0.3% Triton X-100 in PB and applied for 2 h at room temperature. After final washes in PB, samples were mounted in Aqua Poly Mount (Polysciences Eppelheim, Germany) and analyzed with a Zeiss Imager Z1 equipped with an ApoTome and a LSM 701 laser scanning microscope (both Zeiss, Oberkochen, Germany). Projections of picture stacks were calculated with AxioVision 4.8 and Zen software (Zeiss). Note that both ATRAP and ANG II receptor antibodies used here are specific as proven by the negative staining in (*Atrap*^*−/−*^) knockout^6^ and (*Atrap*^*−/−*^*/AT1R-R*^*−/−*^) double knockout mice^30^, respectively.

### Immunoelectron microscopy

We performed immunoelectron microscopy as previously published^50^.Pre-embedding immunoelectron microscopy, staining by antibodies and electron microscopy were carried out as described previously^51–53^. Briefly, retinas were fixed in 4% paraformaldehyde (50 min) and cut into 100 µm thick sections with a vibratome. After blocking, sections were transferred to a solution containing primary antibody and incubated for 4 days (4 °C). After several rinses in PBS, retinal sections were incubated for 2 h at room temperature in biotinylated goat anti-rabbit IgG (1:100; Sigma-Aldrich, Deisenhofen, Germany). Finally the sections were stained using the VectaStain ABC kit (Vector Laboratories, Burlingame, CA) for 1.5 hr at room temperature.

After several washes in PBS and in 0.05 M Tris-HCl (pH 7.6), the sections were transferred to a solution containing 3,39-diaminobenzidine (DAB) [0.05% (v/v) in 0.05 M Tris-HCl, pH 7.6, 10 min], followed by 0.05% (v/v) DAB with 0.03% (v/v) H_2_O_2_. The staining reaction was stopped by Tris-HCl. Subsequently, the sections were rinsed in 0.1 M cacodylate buffer (pH 7.4), post-fixed in 2.5% (v/v) glutaraldehyde in cacodylate buffer (2 hr at 4 °C), and washed in cacodylate buffer overnight at 4 °C. The DAB reaction product was silver-intensified and treated with 0.05% (w/v) gold chloride (Sigma).

The sections were then post-fixed with 2% (w/v) OsO4 in cacodylate buffer for 1 hr, dehydrated in a graded series of ethanol (30–100%), followed by propylene oxide, and flat-embedded in Epon 812 (Serva, Heidelberg, Germany). Ultrathin sections were cut and stained with uranyl acetate. Control vibratome sections lacking primary antibodies were processed as described above. These produced no staining. Ultrathin sections were examined and photographed with a Zeiss EM10 electron microscope.

### Conventional electron microscopy

Sample preparation and image categorization was performed as described earlier^39^: For conventional electron microscopy, retinae were fixed in 4% paraformaldehyde and 2.5% glutaraldehyde for 2 hours at room temperature. Tissue contrasting was carried out by incubation in 4% osmium tetroxide in cacodylate buffer (0.1 M, pH 7.4) for 1.5 hours. Retinae were dehydrated using a rising ethanol series and propylene oxide. The tissue was embedded in Epon resin (Fluka, Buchs, Switzerland). Ultrathin sections (60 nm) were cut and counterstained with uranyl acetate and lead citrate in an automated Leica EM AC20 contrasting system (Leica Microsystems, Wetzlar, Germany). Image acquisition was performed using a Zeiss EM10 electron microscope (Zeiss, Oberkochen, Germany) and a Gatan SC1000 Orius TM CCD camera (GATAN, Munich, Germany) in combination with the Digital Micrograph 3.1 software (GATAN, Pleasanton, CA). Images were adjusted for contrast and brightness using Adobe Photoshop CS6. For the quantification of synaptic ribbon shapes, random images of the outer plexiform layer were taken for each genotype and experimental condition. According to their shape, SRs were classified into rod-, club- and spherical-shaped.

### Ca^2+^ imaging of retinal slices

Ca^2+^ signals from retinal slices were recorded according to the method by Regus-Leidig *et al*.^54^. For Ca^2+^ imaging experiments, sagittal slices of *Atrap*^*+/+*^ and *Atrap*^*−/−*^ mouse retinas were cut at 200 µm with a vibratome (Leica, Mannheim, Germany). Subsequently, slices were incubated for 30–60 min at 37 °C in an atmosphere of 5% CO_2_ / 55% O_2_ with 1 µM Fluo-4 AM (Life Technologies, Grand Island, NY) and 0.5 µl pluronic acid (Life Technologies) in a solution containing (in mM): 117 NaCl, 3 KCl, 2 CaCl_2_, 1 MgCl_2_, 0.4 NaH_2_PO_4_, 25 NaHCO_3_, 15 Glucose (pH 7.4). Following incubation, slices were washed twice and immersed in an extracellular solution containing (in mM): 132 NaCl, 5.4 KCl, 5 CaCl_2_, 1 MgCl_2_, 5 Hepes, 10 glucose (pH 7.4). A Zeiss Examiner D1 microscope (Zeiss, Germany) equipped with a 63x water immersion objective was used to visualize regions of interest (ROIs), and the images were captured by an AxioCam Hsm camera (Zeiss). Small rectangular ROIs (~25 × 5 µm) were placed in the outer plexiform layer. Each region of interest contained several rod and cone terminals, but individual responses of rods and cones could not be distinguished within a single ROI. Therefore, each response measured within a single ROI represents an average of a small sample of both rods and cones. Photoreceptor terminals in the slice preparation were depolarized with a solution containing 150 mM KCl. By application of Nernst’s equation, we calculated the resulting membrane potential to be near 0 mV. The KCl solution was applied to the preparation with a focal perfusion system (ALA Scientific Instruments, Farmingdale, NY) controlled by the Patchmaster software (Heka, Lambrecht, Germany). The tip of the perfusion system was located at a distance of ~500 µm from the tissue and it was operated with minimum pressure to rule out motion artefacts. Under these experimental conditions, a concentration of 150 mM KCl proved to reliably evoke depolarization-induced Ca^2+^ influx. Retinal slices were incubated with 1 µM thapsigargin for 10 min after loading with Fluo-4 AM. Imaging data were acquired with the Axiovision software (Zeiss, Jena, Germany) at frame rates ranging from 5 to 20 Hz. Since ratiometric imaging using Fura-2AM led to increased acquisition rates beyond the physiological time frame of synaptic responses, imaging experiments were carried out with Fluo-4 AM. Data analysis was performed with custom-made scripts using the software packages Matlab (MathWorks, Natick, MA) and Origin (Microcal, Northampton, MA). Time-dependent decrease of mean ΔF/F0 was fitted with a 1st order exponential function using Microcal Origin (Northampton, MA):

$$\frac{\Delta F}{{F}_{0}}={A}_{1}\cdot \exp (\frac{-t}{\tau })+{y}_{0},$$

where A_1_ is the amplitude at t = 0, τ is the time constant of decay, and y_0_ represents an offset.

Rundown of fluorescence in imaging experiments was estimated by repeated application of 150 mmol/l KCl to photoreceptor terminals before pharmacological treatment with isradipine or thapsigargin. The fluorescence signal usually decreased by 10–15% before a constant amplitude was obtained. The peak amplitude of the first KCl-induced Ca^2+^ signal has been used throughout as control.

### Ca^2+^ imaging of cerebellar slices

Using the methods as previously published^55^, Ca^2+^ signals were recorded from mouse cerebellar slices. The *Atrap*^*+/+*^ and *Atrap*^*−/−*^ mice were anesthetized by isofluran before decapitation, and their cerebella were removed from the skull and immersed in an ice-cold, oxygenated (5% CO_2_/95% O_2_, pH 7.4) slice preparation solution containing (in mM) 87 NaCl, 3 KCl, 25 NaHCO_3_, 1.25 NaH_2_PO_4_, 3 MgCl_2_, 0.5 CaCl_2_, 75 sucrose and 25 glucose. Sagittal slices of 300 µm were prepared with a vibratome (Leica VT 1200 S, Leica Instruments, Nussloch, Germany) and transferred to a Nylon basket slice holder for incubation in artificial cerebral spinal fluid (ACSF) containing (in mM) 126 NaCl, 3 KCl, 25 NaHCO_3_, 15 glucose, 1.2 NaH_2_PO_4_, 1 CaCl_2_, and 2 MgCl_2_ at 34°C. The slices were allowed to recover in ACSF with continuous oxygenation for at least 0.5 h.

Before imaging, slices were incubated with 1 µM Fluo-4 AM as described for the retina. Subsequently, the slices were washed twice and immersed in extracellular solution containing (in mM) 126 NaCl, 3 KCl, 25 NaHCO_3_, 15glucose, 1.2 NaH_2_PO_4_, 2.5 CaCl_2_, and 1 MgCl_2_. To depolarize, KCl (150 mM) in extracellular solution was applied to the cerebellar slices for 5 s by a focal custom-made application system. To block sarco-endoplasmic Ca^2+^-ATPase (SERCA) activity, slices were incubated with 5 µM thapsigargin for 5 min after loading with Fluo-4 AM; then 10 mM caffeine was applied for 1 min to deplete intracellular calcium. A Zeiss microscope (Axioskop 2 FS mot, Germany) equipped with a 40x water immersion objective was used to visualize the region of interest and the images were captured by a QuantEM 512SC camera (Photometrics, Tucson). Imaging acquisition was controlled by Imaging Workbench software 5.2.20.6 (INDEC BioSystems, USA) at 20 Hz frame rate. Data analysis was performed with custom-made scripts by Matlab (MathWorks, USA) and Graphpad Prism 5.0 (La Jolla, USA). All data were shown as mean ± SEM. Post-hoc Tukey one-way ANOVA was used for multiple group comparison.

### Statistical analysis

All data were given as mean and standard error of the mean. Experiments were repeated at least five times. All animal data were obtained from 5–6 animals. If not otherwise stated, test for statistical significance was performed by ANOVA. To test variability the Levene test was used. All statistical analyses were performed by GraphPad, Systat, Sigmaplot or Excel. We used ImageJ to quantify the Pearson’s correlation coefficient of immunostaining co-localization.
